# Autoinflammation due to homozygous S208 *MEFV* mutation

**DOI:** 10.1136/annrheumdis-2018-214102

**Published:** 2018-10-24

**Authors:** Ying Hong, Ariane S I Standing, Sira Nanthapisal, Neil Sebire, Stephen Jolles, Ebun Omoyinmi, Ruud HJ Verstegen, Paul A Brogan, Despina Eleftheriou

**Affiliations:** 1 Infection, Inflammation and Rheumatology Section, UCL Great Ormond Street Institute of Child Health, London, UK; 2 Histopathology Department, UCL Great Ormond Street Institute of Child Health, London, UK; 3 Immunology Department, Immunodeficiency Centre for Wales, University Hospital of Wales, Wales, Cardiff; 4 Department of Paediatric Rheumatology, Sheffield Children’s Hospital, Sheffield, UK; 5 ARUK Centre for Adolescent Rheumatology, UCL, London, UK

**Keywords:** *MEFV*, hypereosinophilia, pyrin, autoinflammation

Heterozygous mutations in the *MEFV* gene disrupting the Serine-242 residue in the 14-3-3 binding motif of pyrin cause Pyrin-AssociatedAutoinflammation with Neutrophilic Dermatosis (PAAND).^1–5^ We now describe familial autoinflammation associated with homozygous Serine-208 mutations in *MEFV*, the second crucial phosphorylation site of the pyrin 14-3-3 binding domain.

**Figure 1:**
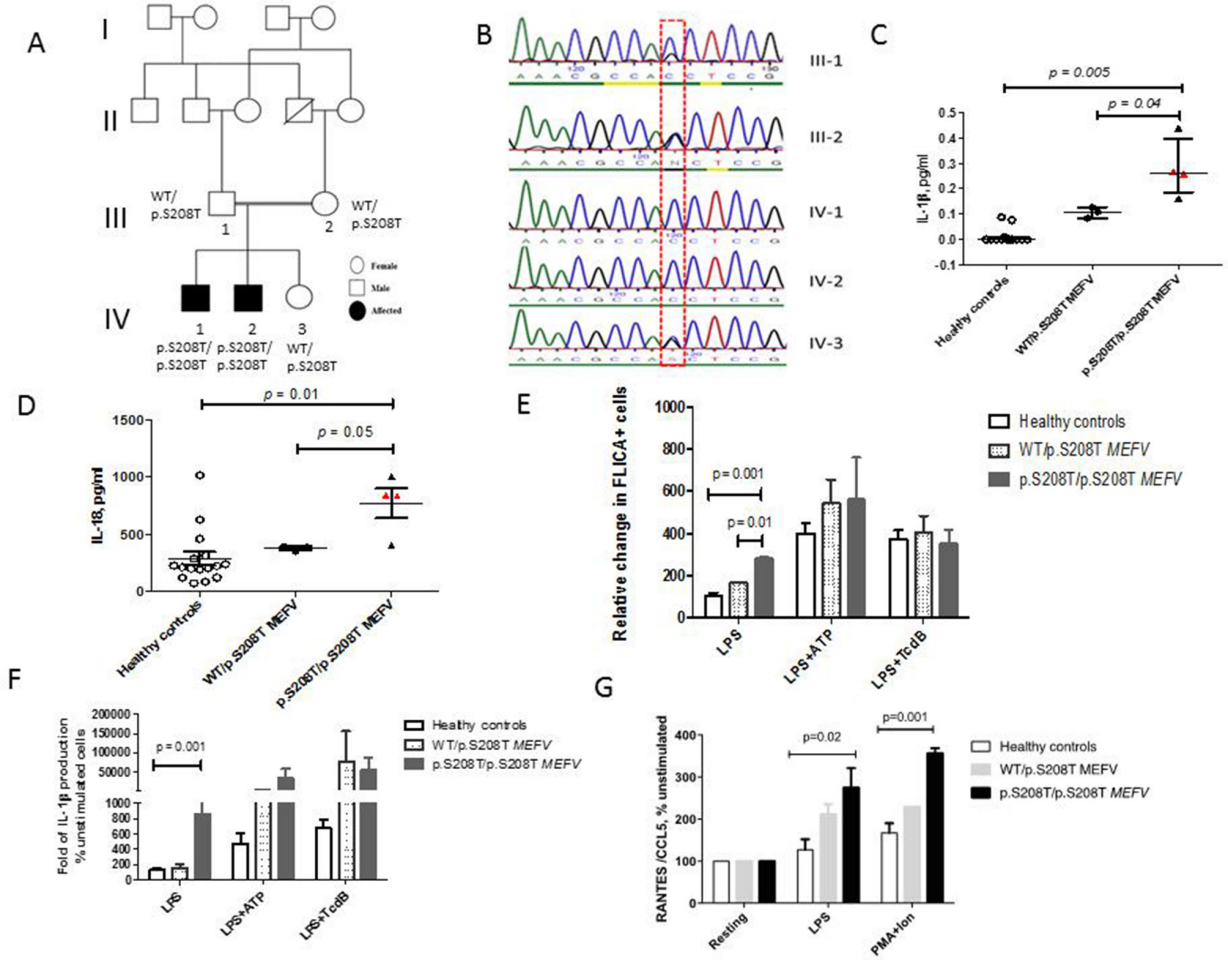
Family tree, genetic sequencing results, proinflammatory cytokines and pyrin inflammasome activation in patients with homozygous p.S208T *MEFV* mutation. (A) The family tree shows the two affected male siblings and their unaffected sibling from the consanguineous marriage of first cousins; segregation of the p.S208T *MEFV* variant is also shown. (B) Sanger sequencing chromatogram of *MEFV* gene aligned to reference sequence exon 2 of *MEFV*. Line indicates a homozygous mutation of *MEFV* at position c.623 (red dashed box) present in both affected patients (IV-1 and IV-2) and heterozygous in parents (III-1 and III-2) and unaffected sister (IV-3). (C) Serum levels of interleukin (IL)-1β were elevated in IV-1 and IV-2 (median 0.26 pg/mL, range 0.16–0.44 pg/mL) compared with healthy controls (median 0 pg/mL, range of 0–0.08 pg/mL, p=0.005) and compared with their unaffected parents (III-2 and III-3), unaffected sibling (IV-3) all with WT/p.S208T MEFV genotype (median levels of 0.1 pg/mL, range of 0.08–0.12 pg/mL, p=0.04). (D) Similar differences were observed for total IL-18 levels that were elevated in IV-1 and IV-2 compared with controls, p=0.01 and compared with III-1, III-2 and IV-3, p=0.05. (E) Monocytes from IV-1 and IV-2 constitutively expressed higher levels of FLICA (caspase-1) in response to lipopolysaccharide (LPS) (mean 398, SEM 84.63) compared with controls (mean 292.8, SEM 95.49, p=0.001) and when compared with heterozygotes for p.S208T *MEFV* mutation (mean 371.5, SEM 109.7, p=0.01). (F) There was an increased release of IL-1β in monocyte supernatants derived from IV-1 and IV-2 following LPS stimulation compared with healthy controls (p=0.001). No significant difference in IL-1β secretion was observed between healthy and patient monocytes after ATP and TcdB addition. (G) There was enhanced release of CCL5 in patient derived peripheral blood mononuclear cells (PBMC) stimulated with LPS (mean relative CCL-5 levels at 233, SEM 37.29 pg/mL) compared with controls (mean relative CCL-5 levels of 126.6, SEM 24.91 pg/mL, p=0.02). Similar differences were observed between groups in CCL-5 release in response to PMA/Ionomycin stimulation, p=0.001. Error bars represent medians and range or for in vitro experiments means+SEM for three biological replicates. P values by Student’s t-test or Mann-Whitney U test <0.05 were considered significant. CCL-5, C-C-motif chemokine ligand 5; FLICA, fluorochrome inhibitor of caspases; PMA, phorbol 12-myristate13-acetate.

Two Pakistani boys (IV-1 and IV-2; figure 1A) born of consanguineous parents presented aged 12 and 9 years old, respectively, with a systemic autoinflammatory disease characterised by a remitting relapsing course over time. Both had recurrent fevers with elevated acute phase responses: C-reactive protein >100 mg/L (reference range (RR)<20); serum-amyloid-A >200 mg/L (RR <10); leucocytosis 92×10^9^/L (RR 4–11; eosinophils 82.4×10^9^/L) and normalisation of these parameters in between fever attacks. Both had recurrent oral ulceration, intestinal inflammation, transient purpuric rashes (leucocytoclastic vasculitis on biopsy), lymphadenopathy (biopsy showed mixed lymphocytic, eosinophil infiltrate), hepatosplemonegaly, arthralgia and failure to thrive. Patient IV-2 developed pulmonary nodular changes and had a history of sterile cutaneous neck abscess at age 5. They had normal complement function studies, immunoglobulin levels and negative autoantibodies. Bone marrow aspirate for IV-2 showed marked eosinophilia (81%) with normal morphology and no malignancy; lymphocyte clonality studies were normal. Digital subtraction angiography and echocardiography were normal. Routine genetic screening for *TNFRSF1A*, *MVK, NLRP3*, *MEFV* exon 10 was wild type. Both patients partially responded to corticosteroids, but subsequently received treatment with cyclophosphamide, mycophenolate mofetil, methotrexate, azathioprine and antitumour necrosis factor alpha therapy. Inflammatory attacks persisted despite these therapies.

Whole exome sequencing and homozygosity mapping (online supplementary text and table S1) identified a homozygous mutation in exon 2 of *MEFV* comprising of a G–C substitution at G623 leading to a serine to threonine substitution at amino acid position 208 (p.S208T) of the pyrin protein. The mutation segregated with disease in a recessive manner (figure 1B) and was not detected in 100 Pakistani healthy controls.

10.1136/annrheumdis-2018-214102.supp1 Supplementary data

We observed elevated levels of circulating IL-1β in patients with homozygous p.S208T *MEFV* mutations compared with healthy controls, p=0.005 and heterozygotes for p.S208T *MEFV* (III-2, III-3, IV-3), p=0.04 (figure 1C). Similar differences between groups were seen for circulating IL-18, p=0.01 (figure 1D). Levels of IL-18 binding protein were not elevated compared with controls, p=0.28 suggesting a contribution to autoinflammation from free IL-18.

Caspase-1 activation, measured as the relative change in fluorochrome inhibitor of caspases (FLICA)^+^ cells after stimulation with lipopolysaccharide (LPS), was significantly elevated in patient CD14^+^cells compared with controls, p=0.001 and with p.S208T *MEFV* heterozygotes, p=0.01 (figure 1E). Healthy control monocytes did not produce significantly increased levels of mature IL-1β in response to LPS likely because of the requirement of a second signal for inflammasome activation to allow cleavage of pro-IL-1β into the mature secreted form. Treatment of healthy control monocytes with LPS and ATP to activate the NLRP3 inflammasome and TcdB (toxin B-positive *Clostridium difficile*), to activate the pyrin inflammasome, resulted in high levels of IL-1β secretion. In contrast, patient monocytes demonstrated increased spontaneous inflammasome activation, because LPS alone was sufficient to induce an increase in IL-1β secretion compared with controls, p=0.001 (figure 1F). No significant difference in IL-1β secretion was observed between healthy and patient monocytes after ATP and TcdB addition or DNA stimulation, indicating similar maximal activities between the dephosphorylated wild-type alleles and the S208T alleles (figure 1F and online supplementary figure 1A). A similar trend was shown for IL-18 release, p=0.03 and with no significant differences seen with addition of ATP and TcdB, p=0.21. In patient cells, there was also increased spontaneous ASC speck formation following treatment with LPS±TcdB compared with control cells.

PBMC with homozygous p.S208T *MEFV* treated with LPS or phorbol 12-myristate13-acetate (PMA)/ionomycin released increased levels of C-C-motif chemokine ligand 5 (CCL5), a potent chemotactic agent for recruitment of eosinophils (p=0.02 and p=0.001, respectively, for comparison with controls, figure 1G). There were no differences in circulating serum CCL-5between patients and controls (p=0.87) possibly as our patients had already received several therapies with normalisation of eosinophil counts at time of sampling.

Another individual, a 2-year-old boy of Pakistani consanguineous descent (online supplementary figure 1B/C for pedigree and Sanger sequencing) was further identified with a homozygous p.S208C (c.A622T) *MEFV* mutation. He presented with a similar phenotype to the above family characterised by recurrent fevers and elevated acute phase responses, oral ulceration (dense eosinophilic infiltration on histology), peripheral blood eosinophilia and osteitis.

This is the first report of human disease associated with mutant S208 *MEFV*, affecting the 14-3-3 protein binding domain of pyrin and leading to constitutive inflammasome activation.

Strong experimental evidence from both animal studies and mutant S208 pyrin transfection experiments in human cell lines indicates a crucial role of this residue for pyrin protein function, with specific clear impact of S208 mutated pyrin on loss of 14-3-3 binding previously documented.^2^ Previous studies have suggested that the S208 mutated pyrin has less of an effect on inflammasome activation compared with mutated S242R pyrin, indicating a functional hierarchy of importance between these two phosphorylation sites.^4^ This may partly explain the recessive mode of inheritance of autoinflammation associated with mutant S208 *MEFV* in contrast to heterozygous mutant S242R *MEFV*.

Hypereosinophilia has not been described in previous reports of PAAND. S208 mutated, constitutively activated pyrin could result in secondary hypereosinophilia as a result of CCL5 production driving eosinophil chemotaxis and/or via primary activation of eosinophils expressing mutated pyrin.^6^ IL-1 blockade may be a successful treatment for these patients and is being considered at the time of writing.
