# Supplementary material for: Autoinflammation due to homozygous S208 MEFV mutation
Source: Ann Rheum Dis. 2018 Oct 24;78(4):571–3. doi: 10.1136/annrheumdis-2018-214102 (PMC6530076; doi:10.1136/annrheumdis-2018-214102)
Supplement: Supplementary data [file annrheumdis-2018-214102supp001.docx]

**Supplemental materials and methods:**

***Ethical approval***

We obtained written informed consent from all the family and controls (ethics approval 08H071382 and 11/LO/0330) who participated in this study.

***Genetic mapping and sequencing***

Homozygosity mapping was performed in all family members for the first family. 200 ng of genomic DNA was isothermally amplified and enzymatically fragmented before hybridization to Illumina Infinium HumanCore Exome Array. These were imaged using the Illumina iScan. Regions of homozygosity were identified using Illumina’s Beadstudio with the loss of heterozygosity detector plug-in (version 1.9.0); the minimum number of contiguous homozygous SNPs was set to 100. Homozygous regions in common in the affected siblings (IV-1 and IV-2) and absent in the unaffected sibling (IV-3) and parents (III-1 and III-2); identified following genotyping on Illumina Human610-Quad arrays are shown below.

| **Chromosome** | **Start** | **Stop** | **Size** |
| --- | --- | --- | --- |
| **3** | 55,542,336 | 74,311,722 | 18,769,386 |
| **3** | 148,415,658 | 154,958,734 | 6,543,076 |
| **6** | 143,743,970 | 147,830,063 | 4,086,093 |
| **15** | 67,547,138 | 68,499,331 | 952,193 |
| **16** | 3,254,247 | 5,289,816 | 2,035,569 |
| **17** | 32,612,687 | 48,351,837 | 15,739,150 |
| **TOTAL** |  |  | **48,125,467** |

Whole exome sequencing (WES) was performed on 50 ng of genomic DNA extracted from peripheral blood. The library preparation for WES was completed with the Illumina Nextera Rapid Capture Exome Library Preparation Kit (Illumina, Inc, San Diego, CA). Sequencing was completed with the Illumina HiSeq 1000 platform. Sequence data were mapped with Galaxy1–3 and human Genome Reference Consortium build 37/hg19 as a reference. Approximately 97.5% of reads were aligned to the reference genome. SNPs were called with wANNOVAR (Wang Genomics Lab,CA)

For the second family, targeted gene panel sequencing and bioinformatic analyses were performed for the index case using standard methodologies, as previously described (Omoyinmi et al, PLoS one 2017).

Sanger sequencing of *MEFV* exon 2 was performed with GAGCAAACGCAGAGAGAAGG for the forward primer and CTTTCCCGAGGGCAGGTA for the reverse primer. FastStart PCR Master Mix (Roche Molecular Systems) was used for amplification, and BigDye Terminator V3.1 Cycle Sequencing Kit (Applied Biosystems) was used for sequencing reaction. Sanger sequencing was performed with the Applied Biosystems 3730 DNA Analyzer, and calls were made with Applied Biosystems 5.2 software. The sequencing output was analyzed with CodonCode Aligner 5.1 (CodonCode Corporation).

***Peripheral blood mononuclear cell (PBMC) isolation and stimulation***

PBMC were isolated from heparinized blood by gradient density centrifugation using Lymphoprep^TM^ (Stemcell^TM^ Technology). Cells were primed with LPS (100 ng/ml) for 4 hrs, then stimulated with 10 mM ATP or 1 mg/ml TcdB for 30 mins to activate pyrin and the inflammasome and with 1µg/ml of poly (dA.dT) with 2.5 µl/ml of Lipofectamine 2000 for 4 hours to induce AIM2 inflammasome activation. To induce production of eosinophil driven cytokines PBMC were stimulated with LPS (100 ng/ml) or PMA (0.05 µg/ml) and Ionomycin (0.5 µg/ml) for 4 hours.

***Measurement of cytokines***

Multiple cytokines were quantified at different time points in the serum of affected patients (IV-1 and IV-2), family members (III-1 and III-2, IV-3), healthy controls, and in supernatants of *in vitro* PBMC culture experiments, by electrochemiluminescence immunoassay using Meso Scale Discovery. Data were analyzed with Discovery Workbench 4.0. IL-18 binding protein (IL-18BP) was measured using ELISA (R&D systems). Control sera was obtained from healthy paediatric controls (n=15, median age 14, range 12-18 years old).

***Caspase-1 activity and apoptosis-associated speck like protein containing a CARD (ASC)-speck formation***

PBMCs were seeded in a 96-well plate at a density of 1.6×10^5^ cells/well (8.0×10^5^ cells/ml). Relevant wells were primed with 100 ng/ml LPS for 4 h, and, if required, then stimulated with 10 mM ATP or 1 mg/ml TcdB for 30 min. Caspase-1 activity was measured using FLICA (ImmunoChemistry Technologies), a cell-permeable fluorescent probe (FAM-YVAD-FMK) that binds active caspase-1. Cells were incubated for 1 h with FLICA at 37°C and stained with PE-conjugated anti-CD14 (BD) to identify monocytes. The gating strategy consisted of including live CD14+ cells, which were subsequently analyzed for the frequency of FLICA+ cells.

For ASC-speck formation cells were fixed with 2% paraformaldehyde and stained for the detection of Apoptosis-associated Speck-like protein containing a Caspase recruitment domain (ASC) specks by Time of Flight Inflammasome Evaluation using the monoclonal anti-ASC antibody and Alexa Fluor 657-conjugated secondary antibodies in combination with DAPI nuclear stain previously described. Subcellular localization of ASC was then analyzed by immunofluorescence.

**Statistical analyses**

All statistics (ANOVA, t test, Kruskal Wallis, and Mann-Whitney-U) and graphs were produced using GraphPad version 5. P < 0.05 was considered significant.


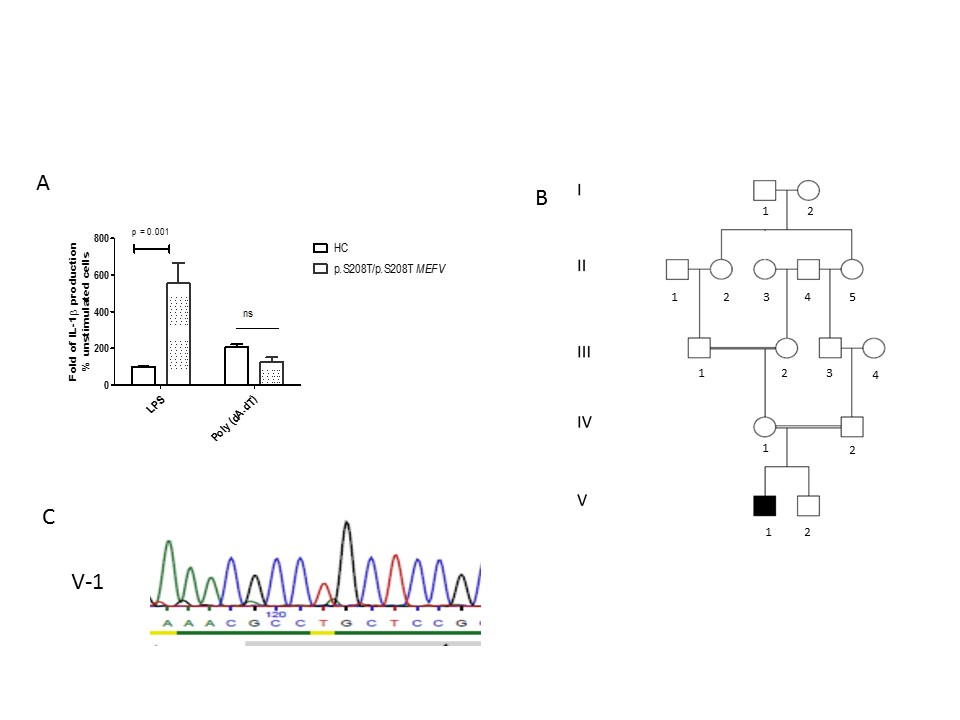


**Supplemental figure 1A:** There was an increased release of IL-1β in monocyte supernatants derived from IV-1 and IV-2 following LPS stimulation compared to healthy controls, p=0.001. No significant difference in IL-1β secretion was observed between healthy and patient monocytes after DNA stimulation with Poly (dA:dT) to induce AIM2 inflammasome activation, p=0.488. B. Family tree for second family where index case (V-1) was a 2 year old Pakistani of consanguineous descent homozygous for p.S208C (c.A622T) *MEFV. C.* Sanger sequencing chromatogram of *MEFV* gene aligned to reference sequence exon 2 of *MEFV* showing homozygous p.S208C *MEFV* mutation in V-1.
